# An uncommon acute type A aortic dissection mimicking an inferior STEMI

**DOI:** 10.11604/pamj.2020.36.247.23821

**Published:** 2020-08-05

**Authors:** Karima Benbouchta, Mehdi Berrajaa, Mohamed Ofkire, Noha El Ouafi, Zakaria Bazid

**Affiliations:** 1 Department of Cardiology, Mohamed I University of Oujda, Mohamed VI University Hospital, Oujda, Morocco

**Keywords:** Acute myocardial infarction, aortic regurgitation, echocardiography, coronary artery

## Abstract

Aortic dissection in the most common fatal disease affecting the aorta. Ascending aortic dissection can lead to coronary malperfusion causing myocardial infarction with ST elevation. The distinction between aortic dissection and a primary myocardial infarction can be difficult because both conditions can have similar presentations. Making the right diagnosis is essential because the therapies used to treat myocardial infarction can be fatal for patients with aortic dissection. Emergency transthoracic echography presents a rapid imaging procedure that provides strong hints of the coexistence of these two diseases, leading to further imaging examination and prevent inappropriate administration of treatments that could cause catastrophic outcome. We report a case of a 62-year-old man admitted to our hospital with chest pain, who was diagnosed as inferior wall myocardial infarction based on electrocardiographic findings. The diagnosis was reassessed due to a significant aortic regurgitation and an intimal tear in the ascending aorta on transthoracic echocardiography. Computed tomography angiogram of the chest and transesophageal echography fully confirmed the presence of ascending aortic dissection. Emergency surgery was successfully performed and the patient recovered well.

## Introduction

Aortic dissection (AD) is the most common fatal disease affecting the aorta [1]. It is due to intimal tear either in the ascending aorta and the aortic arch (Stanford type A), or distal to the origin of the subclavian artery in the descending aorta (Stanford type B) [2]. Sometimes Stanford type A aortic dissection (TAAD) can lead to coronary malperfusion causing myocardial infarction with ST elevation (STEMI). The distinction between AD and a primary myocardial infarction (MI) can be difficult because the condition can have similar presentations. Making the right diagnosis is essential because the therapies used to treat MI can be fatal for patients with AD [3]. Emergency transthoracic echocardiography (TEE) can be simple useful diagnosis tool that could provide strong hints of the coexistence of two diseases. Transesophageal echography (TEE) and computed tomography angiogram (CTA) of the chest and abdomen can confirms the diagnosis with a high sensitivity and specificity [4]. We report the case of a 62-year-old man admitted in our hospital with severe retrosternal chest pain who was diagnosed as STEMI depending on the clinical manifestation and results of the electrocardiogram. A closer inspection of the ascending aorta showed a dissection flap. CTA of the chest and TEE fully confirmed the TAAD. Emergency surgery was successfully performed and the patient recovered well.

## Patient and observation

**Figure 1:**
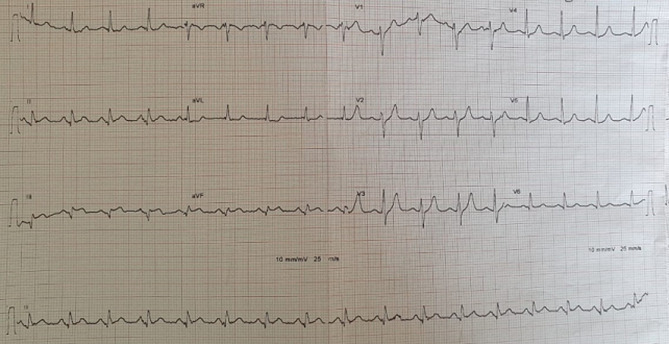
admission ECG showing prominent ST-segment elevations in inferior leads

**Figure 2:**
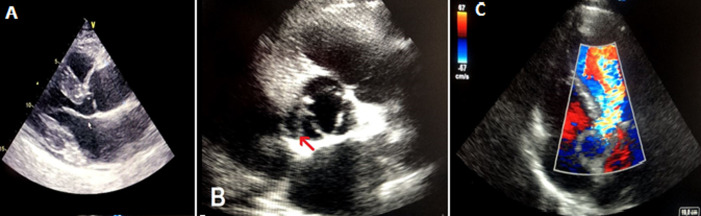
transthoracic echocardiography: (A) a parasternal long-axis view showing dilated aortic root at sinus of Valsalva, left ventricular hypertrophy and small pericardial effusion; (B) a parasternal short-axis view of the aortic valve showing the dissection flap (arrow); (C) apical 5-chamber view of the severe aortic regurgitation in color doppler study

**Figure 3:**
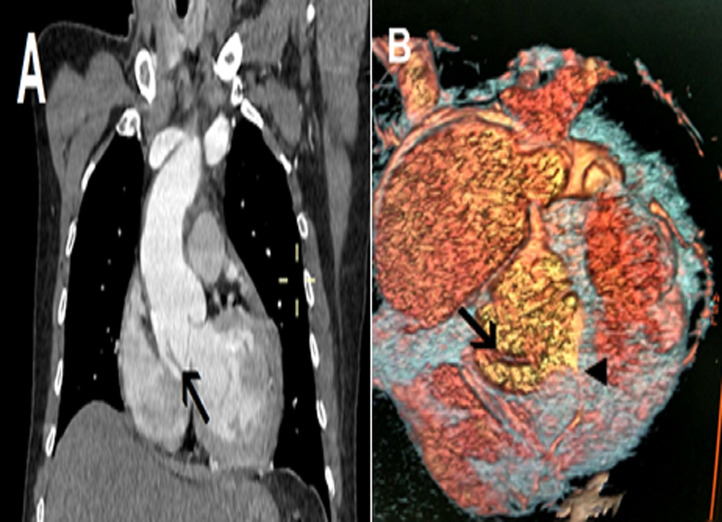
aortic CT angiogram showing Stanford type A aortic dissection: (A) coronal section showing intimal dissection flap (arrow) and pericardial effusion; (B) three dimensionnally reconstructed image of aortic root, showing dissection flap, wich involve the right coronary artery (arrowhead)

**Figure 4:**
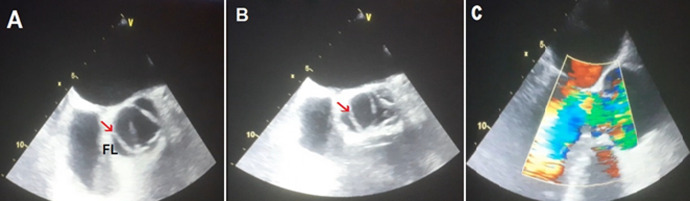
transesophageal echocardiogram: (A) short axis view of the aortic valve in systole showing the dissection flap (arrow) and right coronary artery arising from the false lumen of dissection (FL); (B) in diastole showing the incomplete closure of the aortic valve; (C) long axis view with color doppler demonstrating severe aortic regurgitation

**Figure 5:**
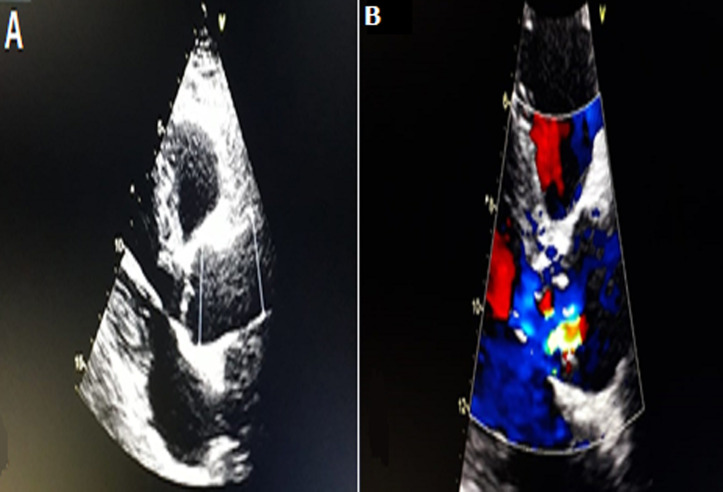
postoperative transthoracic echocardiography: (A) a parasternal long-axis view showing tube graft replacing the ascending aorta; (B) mild valvular AR in color doppler study

A 62-year-old smoker man, with medical history of long-standing uncontrolled hypertension, presented to the emergency department complaining of sudden onset severe retrosternal chest pain since 3 hours, associated with shortness of breath, nausea and sweating. At time of presentation, physical examination showed blood pressure of 180/110 mmHg in the right arm and 170/100 mmHg in the left arm, heart rate of 90 beats per minute, respiratory rate was 18 breaths per minute and oxygen saturartion of 92% breathing ambient air. Cardiac examination showed normal heart sounds, an early diastolic murmur and 4/6 in intensity, best heard on left 3^rd^ intercostal space, with fine crackles in pulmonary auscultation. Admission ECG showed ST-segment elevation in inferior leads suggesting acute inferior myocardial infarction (Figure 1). An urgent bedside transthoracic echocardiogram (TTE) revealed akinesia in the left inferior and lateral walls, a dilated aortic root (42 mm) with evidence of intimal tear in the ascending aorta, a significant aortic regurgitation (AR) with normal appearing aortic leaflets and small pericardial effusion (Figure 2). Based on these findings, ascending AD complicated with inferior MI was highly suspected. In that time, anti-hypertensive therapy was started to reduce systolic blood pressure and thrombolytic treatment was not administered. Emergent CTA of the chest and abdomen was performed and demonstrated a 4.5 cm diameter ascending aorta with a Stanford type A (Debakey type II) acute dissection arising from the level of annulus aorta and a moderate pericardia effusion (Figure 3). TEE was also performed showing ascending AD beginning from sino-tubular junction with severe AR and the right coronary artery (RCA) was arising from the false lumen of dissection (Figure 4). After agreement between the cardiologist, the radiologist, the cardiovascular surgeon and the anesthetist, the diagnosis of dissection of the ascending aorta complicated of MI caused by malperfusion of the RCA and pericardial effusion, was confirmed and the decision for immediate surgical repair was made. The patient underwent valve sparing aortic root replacement successfully, with uncomplicated postoperative course. Echocardiographic evaluation 3 months after surgery showed mild valvular AR with LVEF 50% (Figure 5).

## Discussion

Acute aortic dissection (AAD) is the most common life threatening disease affecting the aorta, with an estimated incidence approximately 5 to 30 cases per million people per year and overall hospital rate of 27.4% [1]. Therefore, early diagnosis and treatment of AD is very important because the mortality rate is 1-2% per hour for the first 24-48 hours from symptom onset [4]. AD can be classified using two classifications based on anatomical, Stanford and Debakey classification. The Stanford classification includes type A and type B where the former involves the ascending aorta and the latter does not. The Debakey classification is divided into three types: I involving the ascending aorta; II it involves only the ascending aorta; and III spares the ascending aorta and arch [5]. The most common underlying comorbidities include hypertension and atherosclerotic disease, and other important risk factors include connective tissue diseases and bicuspid aortic valves [4]. Clinical presentation of AD may be variable and can mimic other more common conditions [1]. Most patients with AD present with chest, or back pain, of sudden onset and usually described as sharp or tearing pain. However, patients may present with symptoms related to AD complications, such as heart failure from acute AR, neurological manifestations, syncope, stroke or other symptoms secondary to vascular insufficiency and malperfusion syndrome [6]. Due to similarities between clinical risk factors and presentations, TAAD may mimic acute MI [7].

Therefore, its diagnostic is difficult. In 25% of the cases, changes in the associated ECG may be compatible with cardiac ischemia leading to a wrong diagnosis, in particular in the case of an elevation of the ST associated with ECG [8]. Acute myocardial infarction (AMI) and AD are both critical illness that require rapid diagnosis and treatment in the emergency department [9]. Rarely, MI can be associated with ascending aortic dissection occurring in 1 to 5% of cases [10]. It is therefore critical to diagnose as early as possible the STEMI that occurs secondary to TAAD because the therapies used to treat MI, can be lethal for patients with AD [3,11]. If patients with AD are given thrombolysis, their mortality rate increases significantly as a consequence of further rupture, expansion and uncontrolled bleeding [12-14]. When AD is complicated by AMI, the pathophysiological mechanisms of coronary malperfusion could be explained by the location where the expanding hematoma causes narrowing of the affected vessel and the location where the dissection flap can partially occlude the ostium of the coronary artery, affecting the blood flow inside the vessel, which may lead to coronary thrombosis and consequent MI [4,15]. In addition dissection can extend directly into the coronary arteries and usually the right coronary artery (RCA) is affected more often than the left artery [15-18]. The higher incidence of involvement of the RCA is due to the fact that dissection come more often from the right anterior aspect of ascending aorta above the right coronary sinus [19].

The diagnosis of STEMI include by Stanford TAAD is difficult and there is no optimal strategy for emergency physicians. However, emergency transthoracic echocardiogram (TTE) can be of great importance to distinguish STEMI caused by TAAD from atherosclerotic etiology. It presents an easy, effective and no invasive tool in the rapid assessment of patients in emergency setting [20]. It can provide information that directly supports the diagnosis as the visualization of an intimal flap or intramural hematoma. In some cases, the detection of aortic regurgitation with normal valve cusps, aortic root dilatation and pericardial effusion, should also raise the possibility of the presence of dissection [3,21]. In this case, emergency TTE allowed us to distinguish between a true STEMI and an AD. While there was some suspicion for AD based on the history and physical examination. Given the clear signs of ischemia on ECG, it was felt that MI was the most likely diagnosis. However with minutes of TEE we were able to rule in AD. Once the diagnosis of AD is suspected, other imaging studies should be performed without further delay. Contrast enhanced CT scanning, magnetic resonance and TEE are all highly accurate techniques that are useful for the confirmation or the exclusion of the diagnosis [4,22].

CTA is an extremely helpful imaging tool to establish the diagnosis quickly due to its good imaging for the aorta and coronary anatomy and also to determine the presence of any associated complications [22]. TEE may also be performed and would represent the subsequent imaging technique for unclear cases of AD, or to complete the diagnosis and also, it provide the surgeon with valuable information such as the severity and mechanisms of AR and help to determine the optimal management of any consequent AR [23,24]. Once the diagnosis of TAAD is confirmed, urgent surgical intervention is required to prevent aortic rupture and related complications associated with the dissection process such as cardiac tamponade [4]. Medical management before surgery is essential. It aims to control hypertension, pulse rate, by using beta-blockers simultaneously with anti-hypertensive therapy and relieve pain bay analgesic therapy [25]. Surgical treatment basically consists in closing the dissection entry port and replacing the ascending aorta and aortic arch if needed and reconstruction of coronary arteries [26]. Repair of the aortic root and preservation of the aortic valve should always be attempted if possible. However, if both aortic root and the aortic valve are diseased, a complete aortic root replacement with a valved conduit is preferred [25].

## Conclusion

Ascending aortic dissection complicated by STEMI is a rare, but extremely serious condition. Making the correct and timely diagnosis a challenge. That is why it is necessary to have a high index of suspicion of AD in cases of acute chest pain with typical ECG changes for acute MI, particularly inferior infarction. Emergency TTE represents an easy and rapid imaging procedure that provides strong hints of the coexistence of both diseases, leading to further imaging examination and prevent inappropriate administration of treatments that could cause catastrophic outcome. Treatment is by surgery. Unfortunately, even with prompt and correct treatment, the mortality rate remains high.
